# Techniques for Repairing Tegmen Defects When the Ossicles Protrude Above the Floor of the Middle Fossa

**DOI:** 10.1055/a-2646-6383

**Published:** 2025-07-16

**Authors:** Jacob Kosarchuk, Maria Majid, Sydney Ho, Kathryn Noonan, Jonathon Sillman, Carl Heilman

**Affiliations:** 1 Department of Neurosurgery, Tufts Medical Center, Boston, Massachusetts, United States; 2 Tufts University School of Medicine, Boston, Massachusetts, United States; 3 Department of Otolaryngology, Tufts Medical Center, Boston, Massachusetts, United States

**Keywords:** encephalocele, CSF leak, tegmen, middle fossa, mastoidectomy

## Abstract

**Objective:**

Occasionally, repair of tegmen defects can be complicated by the ossicular chain protruding above the floor of the middle fossa, which traditionally requires disarticulation and reconstruction of the ossicles to manage. This manuscript describes modifications of previously described techniques to address this surgical problem.

**Design:**

Case series.

**Participants:**

In this case series we present three patients with tegmen defect and encephalocele where the ossicles protruded over the floor of the middle fossa. In one instance, a “manhole cover” was created by concentrically layering hydroxyapatite cement around the tegmen defect and placing a piece of calvarium harvested from the bone flap over the defect. In another case, a “bony igloo” was drilled into harvested bone flap and placed over the defect, effectively creating a neo-epitympanum.

**Main outcome measures:**

Hearing preservation, CSF leak recurrence.

**Results:**

No patients had recurrence of their encephalocele and/or CSF leak. No patients required manipulation of the ossicular chain intraoperatively. Hearing returned to normal in one case. Hearing worsened in one case, thought to be related to injury to the inner hair cells of the cochlear or cochlear nerve. Hearing did worsen in another case, thought to be related to pneumolabyrinth.

**Conclusions:**

The “manhole cover” and “bony igloo” techniques are pragmatic solutions to this rare but complex surgical problem.

## Introduction

Temporal bone cerebrospinal fluid (CSF) leaks and encephaloceles are uncommon entities that are associated with temporal bone trauma, chronic otitis media, idiopathic intracranial hypertension, obesity, and idiopathic causes. 1 2 Dehiscence of the temporal bone can lead to herniation of the dura and temporal lobe into the middle ear, requiring surgical intervention. 3 Patients may present with clear otorrhea or rhinorrhea, a sense of aural fullness, pulsatile tinnitus, hearing loss, and in serious cases seizures or meningitis. 3 4 5

Defects in the temporal bone are most commonly found in the tegmen tympani or anterior or posterior tegmen mastoideum, leading to herniation of tissue into the epitympanic recess of the middle ear, directly above the ossicles. 6 The location of the defect directs selection of surgical approach; historically, the defect can be addressed via the transmastoid approach, the middle cranial fossa approach (MCFA), or a combined approach. Irrespective of surgical approach, the procedure involves resection of the herniated dura and brain tissue; repair of the dura primarily or augmented with dural substitute; and reconstruction of the tegmen defect with bone, cartilage, fascia, muscle, hydroxyapatite cement, or a combination thereof. 7 8

In instances where the ossicles protrude through the epitympanic roof and above the plane of the middle fossa floor, it is challenging to reconstruct the tegmen without disarticulating and reconstructing the ossicular chain or sacrificing hearing. Both unilateral and bilateral ossicular protrusion have been reported. 9 In this manuscript, we present a series of patients who required modification of previously described techniques for repair of the middle fossa floor in order to preserve hearing without manipulating the ossicular chain ( Table 1 ). We believe these modifications represent simple, pragmatic solutions for a challenging surgical problem.

**Table 1:** Patient characteristics, concomitant superior semicircular canal dehiscence (SSCD), approach selection, and method of tegmen defect repair

Illustrative case	BMI	Concomitant SSCD	Approach	Method of coverage
1	32.85	Yes	MCFA	Manhole cover
2	24.08	No	Combined	Manhole cover
3, right side	30	Yes	Combined	Bony igloo
3, left side			MCFA	Bony igloo

Abbreviations: BMI, body mass index; MCFA, middle cranial fossa approach.

## Cases

### Illustrative Case 1

**Fig. 1:**
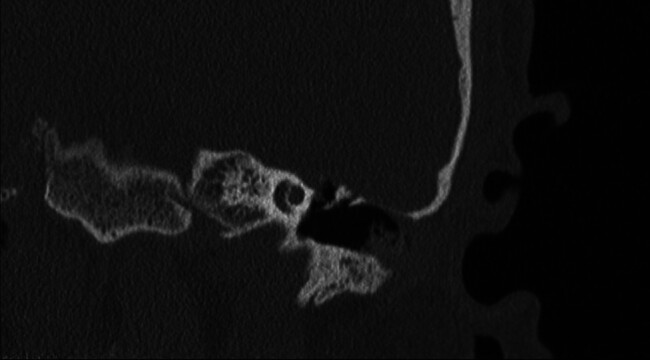
Preoperative computed tomography showing left-sided tegmen defect with the malleus visible over the floor of the middle fossa.

A 51-year-old female with chronic bilateral Eustachian tube dysfunction presented with a 3-year history of bilateral (left greater than right) hearing loss; audiometry showed moderate left-sided conductive hearing loss with an air–bone gap (ABG) of 35 dB. Pure tone audiogram (PTA) was 52.5 dB, and word recognition was 100%. Computed tomography (CT) and magnetic resonance imaging (MRI) showed bilateral tegmen and superior semicircular canal dehiscence with encephalocele involving the malleus and incus on the left ( Fig. 1 ). Surgical repair of the left-sided encephalocele using the MCFA was recommended.

**Fig. 2:**
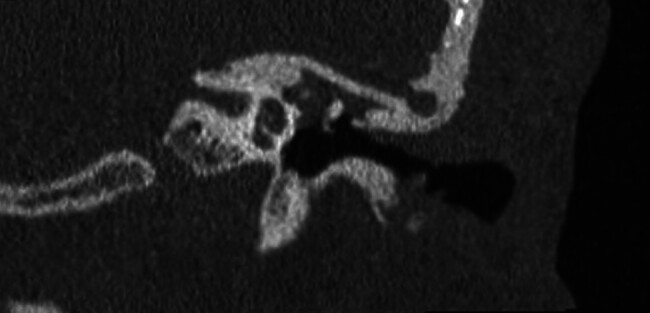
Postoperative computed tomography showing the “manhole cover” method of covering the tegmen defect.

A left temporal craniotomy was performed, with the incision designed such that mastoidectomy and ossicular reconstruction could be performed if deemed necessary at the time of surgery. The tegmen defect was identified during dissection of the dura off the floor of the middle fossa. The mucosa of the mastoid was visible and adherent to the dura, and the malleus was seen to be piercing the dura inferiorly. A small defect in the superior semicircular canal was identified and sealed with wax. The tegmen defect was so large that the facial nerve was exposed from the labyrinthine to the tympanic segment. The dura was then peeled off the greater superficial petrosal nerve (GSPN), middle meningeal artery (MMA) was divided, and then the V3 segment of the trigeminal nerve was exposed. The decision was made to sacrifice GSPN to allow for watertight dural closure around the defect. At this point, the malleus was noted to be projecting above the plane of the tegmen and into the middle fossa. Hydroxyapatite cement was laid circumferentially around the defect. Then a portion of the temporal bone flap was cut to size and laid on the cement like a “manhole cover” and sealed with additional bone cement ( Fig. 2 ). AlloDerm graft was placed extradurally along the floor of the middle fossa, the bone flap was replaced, and the skin was closed in layers.

The patient's recovery was uneventful, and they were discharged on postoperative day 2. Hearing was noted to be stable at 2-month follow-up. Postoperative audiogram showed moderately severe sensorineural hearing loss (SNHL) with closure of the ABG. There was an appreciable interval worsening of pure tones with PTA of 71.25 dB and word recognition decreased to 72%. She unfortunately developed cholesteatoma on the left side that required multiple repairs several years after the index operation.

### Illustrative Case 2

A 48-year-old female presented with 2 years of worsening right-sided pulsatile tinnitus, aural fullness, and intermittent otalgia that were refractory to conservative measures. She was found to have bilateral mastoiditis on CT and MRI, and underwent bilateral myringotomy. The patient's audiogram demonstrated mild right-sided conductive hearing loss with ABG of 12.5 dB, PTA 21.25 dB, and 100% word recognition. Unfortunately, her symptoms worsened, and repeat CT showed tegmen defects with encephalocele herniation onto the ossicles, and operative intervention using a combined approach was recommended.

Mastoidectomy was performed first. The encephalocele was seen extending onto the ossicles through the mastoid antrum and was debrided to minimize trauma to the ossicular chain. This was followed by temporal craniotomy. During dural dissection, several additional small defects were noted in the tegmen mastoideum. The encephalocele was again encountered and divided with bipolar cautery, and the surrounding dura was resected circumferentially. A portion of the craniotomy flap was cut to create a “manhole cover” and was affixed to the floor of the middle fossa with hydroxyapatite bone cement. AlloDerm grafts were placed both intra- and extradurally. The bone flap was then replaced, and the skin closed in layers.

The patient was discharged 2 days post-operation without complication. On follow-up, the patient noted some pulsatile tinnitus on the left, but reported overall subjectively better hearing on the right. Audiometry showed closure of the ABG with no decrease in bone line, representing a return to normal hearing. Word recognition remained 100%. Unfortunately, no postoperative CT was obtained for this patient.

### Illustrative Case 3

**Fig. 3:**
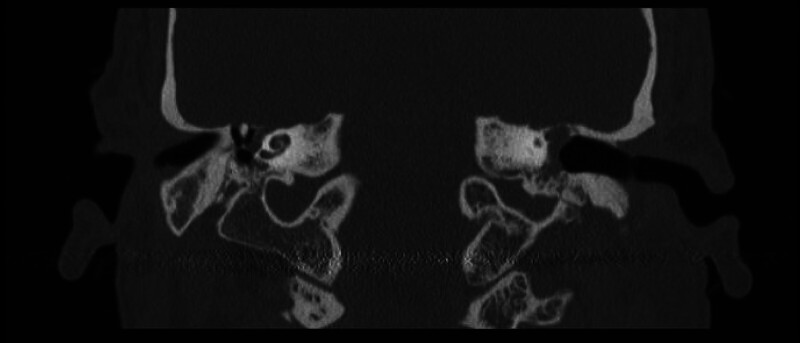
Preoperative computed tomography showing tegmen defect on the right side with the malleus above the floor of the middle fossa.

**Fig. 4:**
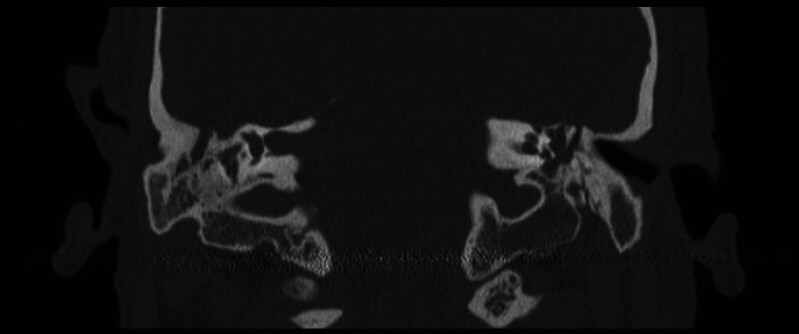
Preoperative computed tomography showing left-sided tegmen defect with the malleus at the level of the floor of the middle fossa.

A 59-year-old male with a history of bilateral hearing loss, Eustachian tube dysfunction, vertigo, and pulsatile tinnitus presented after a CT of temporal bone showed defects of the tegmen mastoideum and tympani with encephalocele and superior semicircular canal dehiscence bilaterally. The ossicles were noted to be projecting above the floor of the middle fossa bilaterally ( Figs. 3 and 4 ). Myringotomy performed in clinic was positive for beta-2-transferrin. Preoperative audiogram revealed bilateral mild conductive hearing loss with ABG of 26.25 and 22.5 dB on the left and right, respectively. Word recognition scores were stable bilaterally at 96%. The decision was made to repair the right side, followed by the left.

**Fig. 5:**
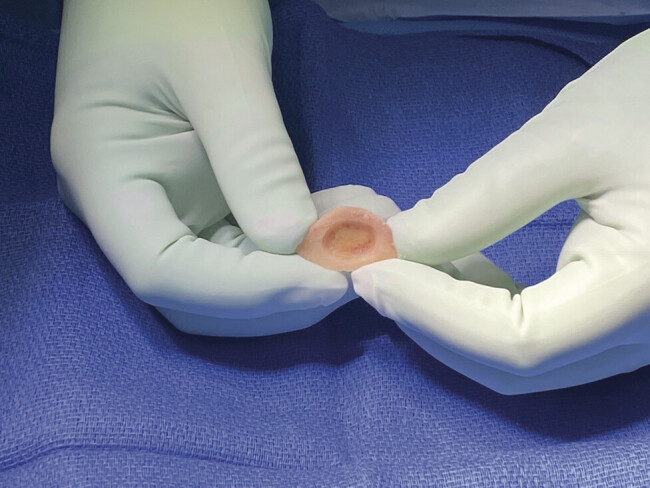
Intraoperative photo showing the “bony igloo” drilled into harvested bone flap for re-creation of the epitympanum.

A combined approach was chosen for the right side, and mastoidectomy was performed first. The encephalocele was seen to be in contact with the ossicular chain, and was debrided prior to performing a right temporal craniotomy. A portion of the superior aspect of the bone flap was separated from the main bone flap, and a diamond burr was used to drill the inner table and marrow from the center of the smaller flap, creating a bony “igloo” ( Fig. 5 ). After dissection of the dura from the floor of the middle fossa, the malleus was noted to be projecting above the defect. The bone flap was placed over the defect, and the ossicles were inspected through the mastoidectomy defect and found to be completely free. AlloDerm grafts were then placed both intra- and extradurally. The bone flap was augmented with titanium mesh and replaced, followed by closure of the skin in layers.

The MCFA was chosen for the left side. A temporal craniotomy was performed, with some additional parietal bone taken for grafting purposes. Similarly to the other side, the center of the smaller bone flap was drilled out to create a bony dome to cover the ossicles. After dural dissection from the floor of the middle fossa, the encephalocele was resected and soft tissue was removed from the middle ear. The malleus was again seen to be protruding over the floor of the middle fossa, and additional defects in the mastoid and superior semicircular canal were identified and sealed with bone wax. The bony igloo was placed over the tegmen defect and sealed with bone cement. AlloDerm was placed intra- and extradurally. The bone flap was augmented with titanium mesh and replaced, followed by closure of the skin in layers.

**Fig. 6:**
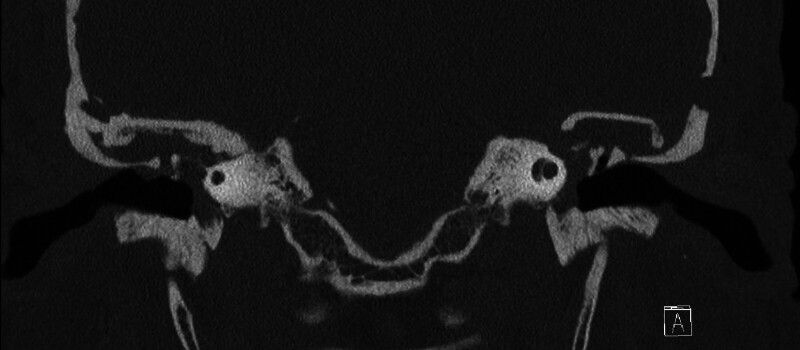
Postoperative computed tomography showing the malleus and tegmen defect covered with a “bony igloo” and AlloDerm.

Postoperatively the patient continued to have mixed hearing loss and tinnitus, right greater than left. The patient had some gait imbalance initially that did improve. Audiogram on the left showed a decline in the higher frequencies with no improvement in ABG. The right side had a decline to moderate mixed hearing loss with no improvement in ABG and decreased bone line. Word recognition remained stable at 96% bilaterally. Postoperative CT showed pneumolabyrinth and bilateral middle ear effusions ( Fig. 6 ), which were proven on myringotomy to be beta-2-transferrin negative. Bilateral tympanostomies were recommended; on the right, the procedure was unable to be performed due to cholesteatoma formation and attic retraction. The left-sided procedure was successful. Importantly, the ossicular chains were examined and found to be intact and mobile.

## Discussion

Temporal bone encephaloceles can be congenital or acquired. Obesity, chronic elevation in intracranial pressure, chronic otitis media, obstructive sleep apnea (OSA), aging, trauma, and iatrogenic causes (e.g. prior mastoid surgery) are identified causes of tegmen defects and encephaloceles. 2 Of note, only obesity was noted as a risk factor for patients in this series (67%, Table 1 ). Not every tegmen defect is associated with encephalocele or CSF leak, and it is thought that concomitant dural pathology is required for encephalocele formation in the setting of tegmen defects. CSF leaks are definitively diagnosed by testing fluid obtained from the ear or nose for beta-2-transferrin, 10 though operative repair without first obtaining testing fluid a beta-2-transferrin is commonly practiced. Additional workup with high-resolution computed tomography (HRCT) or MRI of the skull base is performed if an encephalocele is suspected. 1 9 HRCT can detect multiple defects in the middle fossa floor as well as concomitant superior semicircular canal dehiscence (SSCD). On MRI, herniated brain tissue and CSF signal is readily visible on T2-weighted sequences to help confirm a diagnosis.

The transmastoid approach is well-suited for defects in the tegmen mastoideum, tegmen tympani, or posterior fossa, and requires no brain retraction, but is not appropriate for lesions of the anterior epitympanum with an otherwise normal ossicular chain as this often requires manipulation of the ossicular chain to access the defect. 11 12 The MCFA allows for full visualization of the middle fossa floor, has an excellent success rate, and has good hearing outcomes, but carries the risk of seizures secondary to temporal lobe retraction or injury to the vein of Labbé. Many surgeons prefer a combined approach. The mastoidectomy permits early visualization of the encephalocele and dissection from the ossicular chain while the MCFA provides wide exposure of the floor of the middle fossa, which facilitates more thorough repair of any defects of the tegmen or semicircular canals. 13 The wider exposure afforded by the MCFA also allows for identification of other anterior defects that may not be appreciated by mastoidectomy alone. Once the herniation and defect have been identified, the herniated tissue is amputated, the dura is closed, and the tegmen is reconstructed.

In instances where the malleus protrudes through the tegmen defect above the floor of the middle fossa, it can be challenging to reconstruct the middle fossa floor without having to disarticulate and reconstruct the ossicular chain to preserve hearing. Although there have been significant advances in reconstruction techniques and materials since the procedure was introduced in the 1950s, achieving closure of the ABG has been challenging. 2 Thus, there is value in trying to avoid deconstructing and reconstructing the ossicles during repair of encephalocele and tegmen defect. The techniques described in this manuscript obviate the need for ossicular chain manipulation. In the first case, hydroxyapatite cement was layered concentrically around the tegmen defect to a height that surpassed that of the dehiscent ossicles, followed by placement of autologous bone harvested from the craniotomy flap over the defect. In the third case, the inner table and marrow were drilled out of a portion of the bone flap so that a “bony igloo” was created within the harvested bone, which allowed sufficient space over the exposed ossicles when placing the autograft. In cases utilizing the combined approach, it was possible to verify the freedom of movement of the ossicular chain at the time of surgery.

Our results are summarized in Table 2 . In the first case, the patient's ABG closed but the patient developed an SNHL, likely from injury to the inner hair cells of the cochlea or to the cochlear nerve. The second patient's hearing returned to normal. Unfortunately, the third patient had worsening of hearing bilaterally; the lack of improvement may be related to pneumolabyrinth. Rates of hearing outcomes after tegmen defect repair vary widely in the literature, 14 15 16 and to our knowledge there are no published data that specifically examine hearing outcomes when ossicular reconstruction is required in this disease entity, or if the ossicular chain protrudes above the floor of the middle fossa. No patient had a recurrence of CSF leak or encephalocele. Two of the three patients in this series (67%) developed cholesteatoma requiring surgical intervention. In the first patient (and first case of cholesteatoma), the cholesteatoma developed near the tegmen repair site, and we believe it may be due to epithelium trapped there during the index surgery. Both the patients who developed cholesteatoma also had a documented history of Eustachian tube dysfunction and we hypothesize that the cholesteatoma may be related to postoperative inflammation; to our knowledge there is no literature on the association of comorbid Eustachian tube dysfunction with postoperative cholesteatoma formation, and further investigation is warranted to determine if such a relationship exists.

**Table 2:** Patient hearing outcomes

Patient	Pre-op PTA (dB)	Pre-op air–bone gap (dB)	Pre-op word recognition (%)	Post-op PTA (dB)	Post-op air–bone gap	Post-op word recognition (%)
1	52.5	35	100	71.25	Closed	72
2	21.25	12.5	100		Closed	100
3, right side		22.5	96			96
3, left side		26.25	96			96

## Conclusion

Temporomastoid encephaloceles are an uncommon pathology that can become difficult to manage from a surgical perspective when the ossicular chain protrudes over the plane of the middle fossa floor. We present three methods that utilize hydroxyapatite bone cement and the craniotomy flap to repair these defects without manipulation or reconstruction of the ossicular chain.

## References

[1] Pelosi S Bederson J B Smouha E E Cerebrospinal fluid leaks of temporal bone origin: selection of surgical approach Skull Base 2010 20 04 253 259 21311618 10.1055/s-0030-1249249 PMC3023320

[2] Brackmann D Shelton C Arriaga M Otologic Surgery. 4th ed Elsevier 2015

[3] Wind J J Caputy A J Roberti F Spontaneous encephaloceles of the temporal lobe Neurosurg Focus 2008 25 06 E11 10.3171/FOC.2008.25.12.E11 19035698

[4] Kuhweide R Casselman J W Spontaneous cerebrospinal fluid otorrhea from a tegmen defect: transmastoid repair with minicraniotomy Ann Otol Rhinol Laryngol 1999 108(7 Pt 1):653 658 10435923 10.1177/000348949910800706

[5] Tsalouchidou P E Zoellner J P Kirscht A Temporal encephaloceles and coexisting epileptogenic lesions Epilepsia Open 2023 8 01 113 124 36408781 10.1002/epi4.12674 PMC9977755

[6] Hendriks T Thompson A Boeddinghaus R Tan H EI Kuthubutheen J Radiological findings in spontaneous cerebrospinal fluid leaks of the temporal bone J Laryngol Rhinol Otol 2021 135 05 403 409 10.1017/S0022215121001171 33966670

[7] Glasscock M E III Dickins J RE Jackson C G Wiet R J Feenstra L Surgical management of brain tissue herniation into the middle ear and mastoid Laryngoscope 1979 89 11 1743 1754 502695 10.1288/00005537-197911000-00005

[8] Perez E Carlton D Alfarano M Smouha E Transmastoid repair of spontaneous cerebrospinal fluid leaks J Neurol Surg B Skull Base 2018 79 05 451 457 30210972 10.1055/s-0037-1617439 PMC6133665

[9] Alvi S A Jones J W Lin J Bilateral ossicular head dehiscence into the middle cranial fossa Ann Otol Rhinol Laryngol 2018 127 03 209 212 29313370 10.1177/0003489417751956

[10] Severson M Schaurich C G Strecker-McGraw M K Cerebrospinal Fluid Leak StatPearls Publishing 2024. Accessed September 13, 2024 at:http://www.ncbi.nlm.nih.gov/books/NBK538157/30844184

[11] Kim L Wisely C E Dodson E E Transmastoid approach to spontaneous temporal bone cerebrospinal fluid leaks: hearing improvement and success of repair Otolaryngol Head Neck Surg 2014 150 03 472 478 24395620 10.1177/0194599813518173

[12] Semaan M T Gilpin D A Hsu D P Wasman J K Megerian C A Transmastoid extradural-intracranial approach for repair of transtemporal meningoencephalocele: a review of 31 consecutive cases Laryngoscope 2011 121 08 1765 1772 21647915 10.1002/lary.21887

[13] Carlson M L Copeland W R III Driscoll C L Temporal bone encephalocele and cerebrospinal fluid fistula repair utilizing the middle cranial fossa or combined mastoid-middle cranial fossa approach J Neurosurg 2013 119 05 1314 1322 23889140 10.3171/2013.6.JNS13322

[14] Swanson J Oetojo W Uram Z Treatment of tegmen dehiscence using a middle fossa approach and autologous temporalis fascia graft: outcomes from a single center Clin Neurol Neurosurg 2022 219 107331 35724613 10.1016/j.clineuro.2022.107331 PMC10171465

[15] Hoang S Ortiz Torres M J Rivera A L Litofsky N S Middle cranial fossa approach to repair tegmen defects with autologous or alloplastic graft World Neurosurg 2018 118 e10 e17 29870840 10.1016/j.wneu.2018.05.196

[16] Braca J A III Marzo S Prabhu V C Cerebrospinal fluid leakage from tegmen tympani defects repaired via the middle cranial fossa approach J Neurol Surg B Skull Base 2013 74 02 103 107 24436896 10.1055/s-0033-1333616 PMC3699214

